# Intratumoural Bacterial Co‐Occurrence is Associated With an Altered Tumour Immune Microenvironment and Poor Prognosis in Colorectal Cancer

**DOI:** 10.1002/cam4.72391

**Published:** 2026-10-29

**Authors:** Marina Rubio Garcia, Vicky Bronnec, Maïwenn Kerdreux, William Rosenbaum, Thyra Löwenmark, Anna Löfgren‐Burström, Ingrid Ljuslinder, Sofia Edin, Richard Palmqvist

**Affiliations:** ^1^ Department of Medical Biosciences Umeå University Umeå Sweden; ^2^ Department of Diagnostics and Intervention, Oncology Umeå University Umeå Sweden

**Keywords:** colorectal cancer, microbiota, survival, tumour microenvironment

## Abstract

Alterations in the composition of the gut microbiota have been associated with colorectal cancer (CRC), with growing evidence indicating that specific microbes can have an impact on the tumour microenvironment. In this study, we investigated the associations of an oral pathobiont consortium comprising *P. stomatis, F. nucleatum sensu lato* and *P. micra* with the tumour immune microenvironment and the clinical presentation of CRC patients. Bacterial levels were quantified in fresh frozen tumour tissues using qPCR and the tumour immune environment was analysed using multiplex immunohistochemistry and multispectral imaging, profiling cytotoxic T cells, T‐helper 1 cells, regulatory T cells, B cells, macrophages and regulatory immune checkpoint molecules (CTLA‐4, PD‐1 and PD‐L1). Tumours with polymicrobial intratumoural co‐occurrence were more frequently right‐sided and exhibited microsatellite instability compared with tumours containing low bacterial levels or high levels of *F. nucleatum sensu lato* alone. Furthermore, polymicrobial tumours exhibited an altered tumour immune microenvironment, with decreased infiltration of FOXP3^+^ T regulatory cells and CD68^+^ macrophages, and increased tumour cell expression of PD‐L1 in the tumour centre. Polymicrobial tumours were also associated with poorer patient survival. In conclusion, our findings suggest a link between the co‐occurrence of *P. stomatis* , *F. nucleatum* *sensu lato* and *P. micra* and an altered tumour immune microenvironment in CRC. Elucidating the relationship between bacterial species and immune marker expression may enable the development of improved prognostic and predictive biomarkers, as well as new possibilities for therapeutic intervention.

**Abbreviations**
BLAST: basic local alignment search tool
BRAF: B‐rapidly accelerated fibrosarcoma
CI: confidence interval
CK: cytokeratin
CMS1: consensus molecular subtype 1
CRC: colorectal cancer
CTLA‐4: cytotoxic T‐lymphocyte‐associated protein 4
FFPE: formalin‐fixed paraffin‐embedded
FOXP3: forkhead Box P3
HR: hazard ratio
KRAS: Kirsten rat sarcoma viral oncogene homologue
MSI: microsatellite Instability
MSS: microsatellite Stable
NCBI: National center for biotechnology information
PD‐1: programmed cell death protein 1
PD‐L1: programmed death‐ligand 1
qPCR: quantitative polymerase chain reaction
ROC: receiver operating characteristic
T‐bet: T‐box expressed in T cells
Th17: T helper 17 cells
TMA: tissue microarray
U‐CAN: Uppsala‐Umeå comprehensive cancer consortium

## 1 Introduction

Colorectal cancer (CRC) is the second leading cause of cancer‐related deaths worldwide and the third most common cancer [1]. CRC is a multifactorial disease linked to individual genetic predisposition and environmental factors. Among the latter, changes in the gut microbiota have increasingly been recognised as a contributing factor to CRC development and progression. The gut microbiota is in constant crosstalk with the colorectal epithelium and plays an important role in maintaining gut homeostasis, increasing intestinal epithelial barrier and modulating host immunity. Microbial imbalances in the gut, known as dysbiosis, can promote inflammation and disrupt the gastrointestinal barrier, potentially leading to carcinogenesis [2].

Several in vitro, in vivo and microbiome profiling studies have shown alterations of both the gut microbiota and intratumoural microbiota associated with CRC. A microbial core of bacteria enriched in CRC includes colibactin‐producing *Escherichia coli* (pks+), enterotoxigenic *Bacteroides fragilis* , *Fusobacterium* spp., *Parvimonas micra* , *Peptostreptococcus stomatis, Porphyromonas gingivalis* and *Prevotella* spp. [3, 4, 5]. *Fusobacterium nucleatum* is one of the most studied bacteria in the CRC context. It has been found associated with several aspects of CRC disease progression, including the suppression of NK cell cytotoxicity and T cell activity through binding the bacterial protein Fap2 to the inhibitory immune receptor TIGIT [6]. *F. nucleatum* has also been linked to a poor prognosis [7]. Other opportunistic bacteria such as *P. micra* and *P. gingivalis* are also suggested to negatively impact patient survival [8, 9, 10]. Mechanistically, *P. micra* contributes to colorectal tumourigenesis through enhancing Th17 immune response [8]. *P. gingivalis* has been found to drive colorectal carcinoma progression by activating the NLRP3 inflammasome in the immune microenvironment [11] and *P. stomatis* has been linked to tumour development via activation of the ERBB2‐MAPK pathway [12]. Growing evidence further suggests that the gut and tumour‐associated microbiota may influence the efficacy of immune checkpoint inhibitors. In CRC, *F. nucleatum* and *Peptostreptococcus anaerobius* have been implicated in impaired anti‐PD‐1 responses through modulation of T cell trafficking to the tumour and myeloid‐derived suppressor cells, respectively [13, 14]. However, recent findings suggest that these interactions are context‐dependent, as *F. nucleatum* has also been associated with enhanced immunotherapy responses in CRC, highlighting the complexity of interactions between tumour‐associated microbiota and the immune response [15].

Recently, Galeano et al. demonstrated that the intratumoural microbiota is not randomly distributed, but highly organised in micro‐niches characterised by epithelial and immune cell functions that promote tumour progression and metastasis [16]. Given that the immune response is a key prognostic factor in CRC, any changes are likely to affect the clinical presentation of the disease [17]. Key negative regulators of the immune response, particularly of T cell activity, include FOXP3^+^ T regulatory cells (Tregs) and the immune checkpoint molecules CTLA‐4, PD‐1 (PDCD‐1) and PD‐L1 (CD274), which are important mediators of tumour immune evasion. PD‐L1 may be directly expressed by tumour cells, and the interaction of PD‐L1 with T cells expressing the ligand PD‐1 negatively regulates the T cell response [18]. Previous studies have also shown *P. stomatis, F. nucleatum, P. micra* and *P. gingivalis* to be associated with the CMS1/MSI immunogenic subtype of CRC [9, 10, 19, 20].

*P. stomatis, F. nucleatum sensu lato, P. micra* and *P. gingivalis* are oral bacteria, suggesting the oral‐gut microbiome axis may play a critical role in CRC through the translocation of oral pathobionts to the gut. While the roles of *P. stomatis, P. micra* and *P. gingivalis* remain less well characterised, *F. nucleatum* has been extensively studied in disease progression and has long been recognised as enriched in CRC patients. With advances in bacterial characterisation, the taxonomy of *F. nucleatum* has recently been revised. The four subspecies previously grouped within the *F. nucleatum* group ( *F. nucleatum* subsp. *nucleatum*, *F. nucleatum* subsp. *animalis*, *F. nucleatum* subsp. *vincentii* and *F. nucleatum* subsp. *polymorphum*) have been reclassified as distinct species named *F. nucleatum, F. animalis*, *F. vincentii* and *F. polymorphum* [21]. This reclassification has introduced ambiguity in the literature regarding whether *F. nucleatum* refers to the species *sensu stricto* or *sensu lato*. In this work, we use *F. nucleatum sensu lato* to refer to this group of four species, while retaining the historical classification when discussing previous studies, where *F. nucleatum* could describe either *F. nucleatum sensu lato* or *F. nucleatum sensu stricto*.

In this study, the aim was to investigate the associations between the oral pathobiont consortium formed by *P. stomatis, F. nucleatum sensu lato, P. micra* and *P. gingivalis* and the tumour immune microenvironment in CRC. Bacterial levels were analysed by qPCR, and the tumour immune contexture was analysed using multiplex immunohistochemistry and multispectral imaging. Multiple immune populations were identified, including cytotoxic T cells, T‐helper 1 cells, regulatory T cells, B cells and macrophages, as well as the expression of the regulatory immune checkpoint molecules CTLA‐4, PD‐1 and PD‐L1. Understanding the possible link between bacterial species and immune marker expression could lead to improved prognostic and predictive biomarkers, as well as new possibilities for therapeutic intervention.

## 2 Material and Methods

### 2.1 Patient Cohort and Sample Collection

The present study included patients from the Uppsala‐Umeå Comprehensive Cancer Consortium (U‐CAN). The project is a longitudinal collection cohort of patient samples including blood, tissue and faeces, as well as radiological and clinical data from patients with CRC, among other cancer types.

The U‐CAN study cohort used in this study has been previously described [9], and included 257 Stage I–IV CRC patients handled at Umeå University hospital with faecal samples collected at the time of diagnosis between the years 2010 and 2014. Stool samples were self‐collected by patients at home prior to the initiation of cancer therapy as previously described [22]. From these study patients, fresh frozen tumour tissue was obtained from 115 surgically resected CRC patients. Pre‐operative radiotherapy was given to 112 (43.6%) of rectal cancer cases.

A tissue microarray (TMA) was assembled utilising retrospectively obtained formalin‐fixed paraffin‐embedded (FFPE) resected tumour specimens from 151 patients, as previously described [23]. In brief, the TMA was generated using the TMA GRAND Master instrument (3DHistech) with a 1 mm punch. For each patient, two cores were taken from the tumour front and one from the centre.

### 2.2 Primer Design for the *P. stomatis* qPCR Assay

SpeciesPrimer BioTool [24] was used for the screening of species‐specific target regions of *P. stomatis*, generating a list of 30 sequences potentially specific to the bacterium. Candidate sequences were further evaluated *in silico* by BLAST (Basic Local Alignment Search Tool) against *Peptostreptococcus* spp. and other related taxa to exclude cross‐reactive regions and to identify targets conserved across available *P. stomatis* genomes on NCBI RefSeq Database (7 genomes available in September 2023 at contig and scaffold level). Specificity was re‐evaluated using updated database content (21 genomes available in November 2025 also at contig and scaffold level). Two sequences meeting both the specificity and intraspecies conservation criteria were selected for qPCR primers (forward and reverse) and probe design using Primer 3Plus. A third candidate from the literature was included in the qPCR optimisation study [3]. Primer sequences are listed in Table 1.

**TABLE 1:** qPCR primers and probes for detection of *P. stomatis* . Forward (F), reverse (R) and probe (P) sequences are shown. The probe is labelled with FAM (fluorescein amidites) and BHQ (Black Hole Quencher).

Primer name	Primer sequences	Amplicon size (bp)	References
Osman	F: 5′ CGGCAGCAGGATACATAGC 3′ R: 5′ TGGACAAGGAGTGGTAGGTT 3′ P: FAM‐TCCCTATAGGACCCCCAAAC‐BHQ‐1 3′	136	Osman et al. (2021) [3]
g94	F: 5′ CCATAAGCTTCAGATTGTCCCTAGA 3′ R: 5′ GGGATAGGTATGTTGAAGACTACGG 3′ P: FAM‐GCCTGCCTGCTAAGCTTGTA‐BHQ‐1 3′	114	In‐house design (2024)
serS	F: 5′ TGTTTCTATTGGCGATGAAAGGA 3′ R: 5′ GCTTAAGGGCTTGGGTTCTAAG 3′ P: FAM‐TGCTGGTCAAGGAAGAAACC‐BHQ‐1 3′	116	In‐house design (2024)

### 2.3 Bacterial Strains

The reference strain *P. stomatis* DSM 17678 and its already extracted DNA were purchased (DSMZ, Leibniz Institute), and four clinical isolates were kindly provided by Dr. Nelly Romani Vestman and Dr. Olena Rakhimova (Department of Odontology, Umeå University). Genomic DNA was extracted from reference and clinical strains using the DNeasy Blood & Tissue kit (Qiagen) with modifications including enzymatic and mechanical lysis, as previously described [25]. After elution, DNA concentration was measured using NanoDrop (DeNovix DS‐11 Spectrophotometer/Fluorometer).

### 2.4 qPCR Optimisation for Primer Selection

The annealing temperature of the three primer sets was tested by qPCR using the QuantStudio 6 Flex Real‐Time PCR system in a 20 μL reaction volume with SYBR Green qPCR Master Mix. Running settings were as follows: an initial denaturation at 95°C for 15 s, an annealing temperature gradient ranging from 56°C to 64°C for 60 s to assess optimal annealing temperature, and a dissociation step at 95°C for 15 s. Melt curve analyses and separation on a 2% agarose gel revealed one specific gene product. Optimal annealing temperature was set to 60°C.

Primer specificity was tested using DNA from *P. stomatis* DSM 17678 and clinical isolates, and assay performance was evaluated across a range of DNA concentrations by qPCR. Reactions were performed on the QuantStudio 6 Flex Real‐Time PCR system in a 20 μL reaction volume using TaqMan Universal Master Mix. The cycling programme consisted of an initial hold step at 50°C for 2 min, followed by an activation step at 95°C for 10 min, and 40 cycles of 95°C for 15 s and 60°C for 1 min. The most suitable primer set was selected based on its ability to target a higher number of *P. stomatis* strains at lower DNA concentrations. The serS primer set was therefore chosen, as it successfully targeted all five *P. stomatis* strains tested, whereas Osman and g94 primers targeted only four.

### 2.5 qPCR Analysis of *P. stomatis* in Fresh Frozen Tissues and Faecal Samples

DNA from tumour tissue and faeces had previously been extracted from the study patients [9]. The cohort included a total of 115 fresh frozen tumour tissue samples and 257 faecal samples. Two tissue samples and 26 faecal samples were excluded due to insufficient DNA quantity. Samples were analysed in duplicates over 40 cycles. Cq values above 38 were considered negative. Duplicates with a difference in SD > 0.5 for Cq values were re‐run in triplicates. If results were still inconclusive, samples were excluded from the analyses. Six fresh frozen tissue samples and three faecal samples were excluded based on this criterion, leaving in total data on *P. stomatis* in tumour tissue samples of 107 patients and faecal samples of 228 patients. Levels of *P. stomatis* were presented as a relative quantification using the 2^−ΔCq^ method. The human gene *PGT* was used as a reference for fresh frozen tissue and the 16S rRNA gene was used as a reference for faecal samples.

*F. nucleatum sensu lato, P. micra* and *P. gingivalis* were previously quantified by qPCR in fresh frozen tissue and faecal samples from this cohort as described elsewhere [9, 10, 22]. The *F. nucleatum* assay was run prior to the recent taxonomic revision and therefore does not distinguish between the species within the former *F. nucleatum* group and detects *F. nucleatum sensu lato* (i.e., *F. animalis, F. nucleatum, F. vincentii* and *F. polymorphum*, Figure S1). In this study, *F. nucleatum* *sensu lato* is collectively referred to as *Fuso4* for simplicity in figures and analyses, as previously described [26].

Relative levels of *P. stomatis, Fuso4* and *P. micra* were stratified into high‐level and low‐level groups using Youden's index analysis, based on receiver operating characteristic (ROC) curve analysis for cancer‐specific survival. Levels of *P. gingivalis* were analysed as positive or negative due to low detection frequency.

A total of 104 patients with complete data on *P. stomatis* , *Fuso4* and *P. micra* were further divided into three groups according to intratumoural bacterial levels, due to the significant associations observed among these species. *P. gingivalis* was not included in subsequent analyses as it showed no clear association with the other CRC‐associated bacteria.

Co‐occurrence groups were created as follows: (i) Low, (ii) *Fuso4* high only and (iii) Polymicrobial high. Group (i) describes cases with low intratumoural abundance of *P. stomatis, Fuso4* and *P. micra* . Group (ii) defines cases with high intratumoural levels of *Fuso4* only, and group (iii) describes cases with high levels of *Fuso4* together with high levels of *P. stomatis* and/or *P. micra* . Two cases exhibited high intratumoural levels of *P. stomatis* only, and one case showed high levels of *P. stomatis* and *P. micra* without concomitant elevation of *Fuso4*. As these patterns did not conform to the predefined group classifications and were too few in number to permit meaningful subgroup analysis, these cases were excluded from further analyses.

### 2.6 Analyses of the Tumour Immune Microenvironment

The assembled TMA, comprising samples from 151 patients, has previously been used to assess the immune response [23, 27]. The analyses were performed through multispectral imaging (VECTRA 3 system, Akoya Biosciences), using two different multiplex immunohistochemistry panels. Markers included in *Panel I* were CD8 (cytotoxic T cells), T‐bet (T‐helper 1 cells), FOXP3 (T regulatory cells), CD20 (B cells), CD68 (macrophages) and cytokeratin (CK). *Panel II* included the immune checkpoints CTLA‐4, PD‐L1, and PD‐1, together with FOXP3 and CK. Since FOXP3^+^ T regulatory cells constitutively express CTLA‐4 [28, 29, 30], CTLA‐4^+^ cells co‐expressing FOXP3 were considered regulatory T cells, as defined in *Panel I*. In contrast, CTLA‐4^+^/FOXP3^−^ cells were analysed separately to capture other CTLA‐4‐expressing T cell populations. For immune cell analyses, preoperatively irradiated rectal cancers were excluded due to the known remodelling effect by radiotherapy on the tumour immune microenvironment [31].

## 3 Statistical Analysis

Statistical analyses were performed using IBM SPSS Statistics 30.0. Associations between categorical variables were assessed using Fisher's Exact test for two groups and Fischer's Freeman Halton Exact test for more than two groups. Correlations between continuous variables were analysed by Spearman's rank correlation. Differences in continuous variables between groups were tested using the Mann–Whitney *U* test for two groups and the Kruskal‐Wallis test for three or more groups.

In cancer‐specific survival analyses, patients were followed from the date of surgery until death or the end of follow‐up (October 2021). Cancer‐specific survival was defined as death with disseminated or recurrent CRC. Kaplan–Meier plots were used to estimate cancer‐specific survival over time, and differences between groups were assessed using the log‐rank test performed on data truncated at 5 years to satisfy the assumption of proportional hazards.

The association of bacterial abundance with cancer‐specific survival was evaluated using univariable and multivariable Cox proportional hazards models. Multivariable analysis was performed using a backward stepwise conditional approach to identify independent predictors of survival.

## 4 Results

### 4.1 *P. stomatis* Shows Strong Associations With Other CRC‐Associated Bacteria in CRC Patients

Levels of *P. stomatis* were quantified by qPCR in 107 fresh frozen tumour tissues and 228 faecal samples from a cohort of 257 patients with CRC. A total of 22 (20.6%) of the tumour tissues were positive for *P. stomatis*, whereas 111 (48.7%) of the faecal samples were positive.

The co‐occurrence of *P. stomatis* with other oral CRC‐associated bacteria was further explored (Table 2). *P. stomatis* was found highly positively associated with *Fuso4* and *P. micra* in both fresh frozen tumour tissue samples and faecal samples (Table 2). *P. gingivalis* was detected in only a small subset of tumours and showed no positive association with any of the other three bacteria in either fresh frozen tumour tissue or faecal samples (Table 2). Therefore, *P. gingivalis* was not included in subsequent analyses.

**TABLE 2:** Associations between *P. stomatis, Fuso4, P. micra* and *P. gingivalis* in fresh frozen tumour tissue and faeces from CRC patients.

		Fresh frozen tumour tissue			Faeces		
		*P. stomatis*			*P. stomatis*		
		Low	High	*p*	Low	High	*p*
*Fuso4*, *n* (%)	Low	66 (75.0)	3 (16.7)	< 0.001*	102 (64.2)	13 (19.7)	< 0.001*
	High	22 (25.0)	15 (83.3)		57 (35.8)	53 (80.3)	
*P. micra* , *n* (%)	Low	80 (90.9)	3 (17.6)	< 0.001*	115 (71.0)	8 (12.1)	< 0.001*
	High	8 (9.1)	14 (82.4)		47 (29.0)	58 (87.9)	
*P. gingivalis* , *n* (%)	Negative	85 (95.5)	15 (83.3)	0.091	158 (97.5)	61 (92.4)	0.125
	Positive	4 (4.5)	3 (16.7)		4 (2.5)	5 (7.6)	

*Note:* The relative levels of *P. stomatis, Fuso4* and *P. micra* were stratified into high‐level and low‐level groups using Youden's index analysis, based on ROC curve analysis for cancer‐specific survival. Levels of *P. gingivalis* were analysed as positive or negative due to low detection frequency. Fisher's exact test was used to compare categorical variables.

Abbreviation: *Fuso4*, *F. nucleatum* *sensu lato*.

* *p* < 0.05.

### 4.2 Bacterial Co‐Occurrence Is Associated With Clinicopathological and Molecular Characteristics in CRC Patients

Due to the strong association between *P. stomatis* , *Fuso4* and *P. micra* in tumour tissue, we classified the cases into three distinct bacterial co‐occurrence groups: Low (*n* = 65), *Fuso4* high only (*n* = 14) and polymicrobial high (*n* = 22) (Table 3). Bacterial co‐occurrence was found associated with female sex, right‐sided tumour location and an MSI phenotype, with the most prominent effects observed in the polymicrobial high group (Table 3). No associations were observed with age, tumour stage, tumour grade or mucinous histological type.

**TABLE 3:** Associations between intratumoural bacterial co‐occurrence and clinicopathological and molecular characteristics in CRC patients.

	Bacterial co‐occurrence in tumour tissue			
	Low	*Fuso4* high only	Polymicrobial high	*p*
Sex, *n* (%)				
Male	40 (74.1)	9 (16.7)	5 (9.3)	0.005*
Female	25 (53.2)	5 (10.6)	17 (36.2)	
Age, *n* (%)				
≤ 59	6 (35.3)	3 (17.6)	8 (47.1)	0.061
60–69	26 (78.8)	4 (12.1)	3 (9.1)	
70–79	25 (67.6)	5 (13.5)	7 (18.9)	
≥ 80	8 (57.1)	2 (14.3)	4 (28.6)	
Localisation, *n* (%)				
Right colon	11 (36.7)	8 (26.7)	11 (36.7)	0.006*
Left colon	15 (78.9)	1 (5.3)	3 (15.8)	
Rectum	39 (75.0)	5 (9.6)	8 (15.4)	
Stage, *n* (%)				
I	22 (73.3)	5 (16.7)	3 (10.0)	0.453
II	21 (67.7)	3 (9.7)	7 (22.6)	
III	16 (53.3)	5 (16.7)	9 (30.0)	
IV	5 (55.6)	1 (11.1)	3 (33.3)	
Mucinous type, *n* (%)				
No	54 (65.9)	11 (13.4)	17 (20.7)	0.588
Yes	9 (52.9)	3 (17.6)	5 (29.4)	
Tumour grade, *n* (%)				
Low	53 (67.1)	12 (15.2)	14 (17.7)	0.146
High	10 (50.0)	2 (10.0)	8 (40.0)	
*KRAS*, *n* (%)				
Wild type	40 (63.5)	8 (12.7)	15 (23.8)	0.794
Mutant	25 (65.8)	6 (15.8)	7 (18.4)	
*BRAF*, *n* (%)				
Wild type	57 (69.5)	11 (13.4)	14 (17.1)	0.036*
Mutant	8 (42.1)	3 (15.8)	8 (42.1)	
MMR status, *n* (%)				
MSS	61 (70.1)	12 (13.8)	14 (16.1)	0.002*
MSI	4 (28.6)	2 (14.3)	8 (57.1)	
Pre‐operative radiotherapy, *n* (%)				
No	42 (58.3)	12 (16.7)	18 (25.0)	0.166
Yes	23 (79.3)	2 (6.9)	4 (13.8)	

*Note:* Fisher's exact test was used to compare categorical variables.

Abbreviation: *Fuso4*, *F. nucleatum* *sensu lato*.

* *p* < 0.05.

### 4.3 CRC Patients in the Polymicrobial High Group Exhibit Worse Cancer‐Specific Survival

Kaplan–Meier plots were generated to compare differences in cancer‐specific survival among the bacterial co‐occurrence groups. The polymicrobial high group was found significantly associated with decreased 5‐year cancer‐specific survival (Figure 1). The prognostic significance of bacterial co‐occurrence remained significant in a multivariable model including tumour stage (HR 4.46, CI 1.38–15.2, *p*‐value 0.017) (Table S1).

**FIGURE 1:**
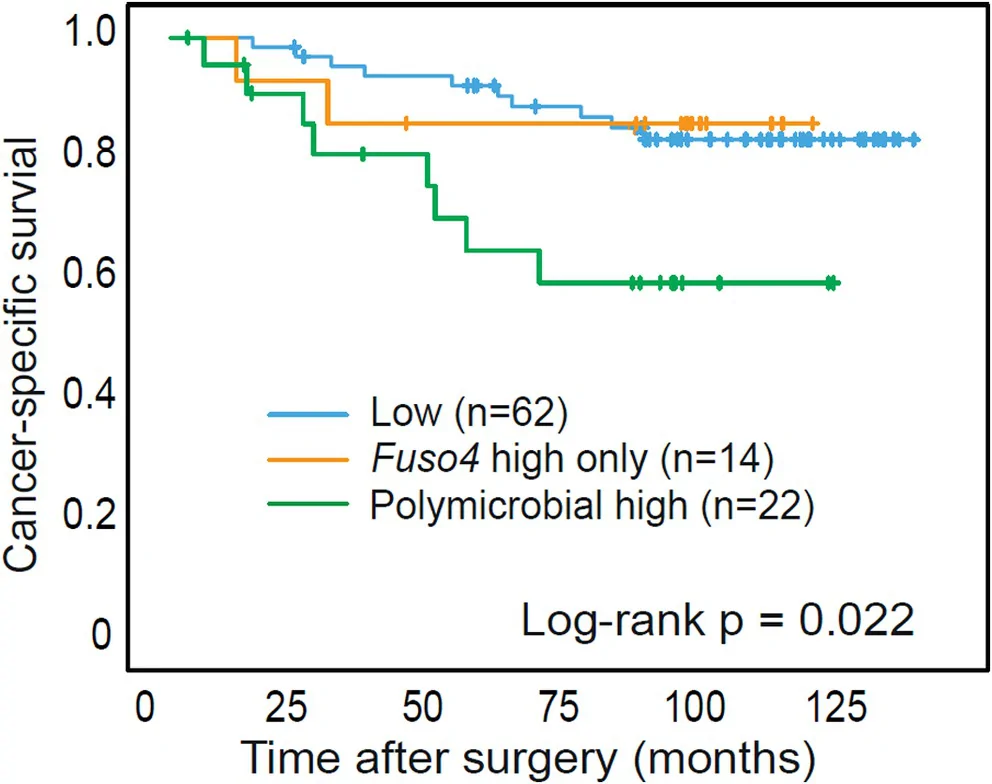
Survival of CRC patients according to bacterial co‐occurrence. Shown is a Kaplan–Meier plot of cancer‐specific survival in CRC patients stratified into three groups: low levels of bacteria, *Fuso4* high only and polymicrobial high. The difference in 5‐year cancer‐specific survival was assessed using the log‐rank test. A *p*‐value < 0.05 was considered statistically significant.

### 4.4 Bacterial Co‐Occurrence Is Associated With Alterations in Tumour Immune Expression Patterns

Tumour infiltration of immune markers was previously assessed in this cohort by multiplex immunohistochemistry and quantified as the number of positive cells per mm^2^ of tumour area, investigating both the stromal and the tumour epithelial area in both the tumour front and centre (Figure 2a), as previously described [23, 27]. Analysed markers included CD8, T‐bet, FOXP3, CD20, CD68, as well as the immune checkpoints CTLA‐4, PD‐L1 and PD‐1. The immune markers were assessed within the three bacterial co‐occurrence groups (Figure 2b). Representative images of multiplex staining according to bacterial co‐occurrence are provided in Figure S2.

**FIGURE 2:**
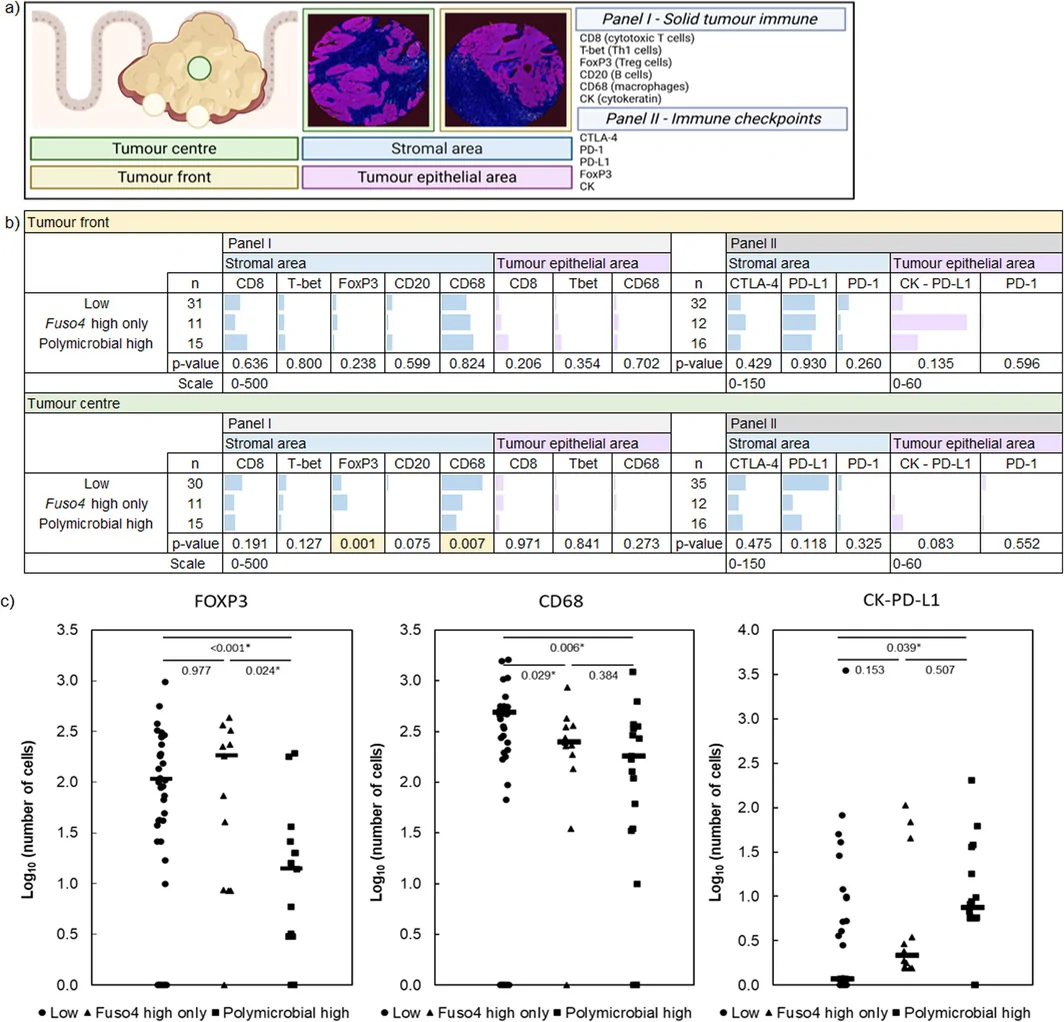
Expression of immune markers in the three bacterial co‐occurrence groups (low, *Fuso4* high only, and polymicrobial high) in tumour tissue of CRC patients. (a) Illustration of the evaluation of immune markers by multiplex immunohistochemistry and multispectral imaging on TMAs derived from the front and centre of CRC tumours, with separate assessment of stromal and tumour epithelial areas. (b) Displays median numbers of positive cells/mm^2^, represented on indicated scales, for the specified immune markers in the stromal or tumour epithelial area of the tumour front and centre, stratified by bacterial co‐occurrence. Differences were assessed by the Kruskal‐Wallis test. In *Panel II*, CTLA‐4 refers to CTLA‐4^+^/FOXP3^−^ cells, whereas data from FOXP3^+^ cells are presented in *Panel I*. (c) Scatter plots displaying the distribution of FOXP3, CD68 and CK‐PDL‐1 positive cells, as indicated, according to bacterial co‐occurrence. Shown are log_10_ values for FOXP3 or CD68 positive cells/mm^2^ infiltrating the stromal area of the tumour centre, and for tumour cells/mm^2^ in the tumour centre expressing CK‐PD‐L1. Horizontal lines represent median number of cells/mm^2^. Differences between groups were assessed using the Mann–Whitney test. *Indicates statistically significant *p*‐values < 0.05. *Fuso4*, *F. nucleatum* *sensu lato*. Created in part with BioRender.com.

Significant differences in immune cell infiltration were observed predominantly in the tumour centre. Specifically, cases in the polymicrobial high group exhibited significantly decreased numbers of infiltrating FOXP3^+^ cells and CD68^+^ cells compared to those in the low group (Figure 2b,c). For FOXP3, a significant difference was also observed between the polymicrobial high and *Fuso4* high only group, indicating an added impact of bacterial co‐occurrence (Figure 2c). In contrast, CK‐PD‐L1^+^ cells in the tumour centre were significantly increased in the polymicrobial high group compared to the low group (Figure 2c). No significant associations were found between bacterial co‐occurrence and other immune markers, such as CD8, CD20, T‐bet or CTLA‐4 (Figure 2b). No associations were found in any of the groups in the tumour front (Figure 2b).

## 5 Discussion

Changes in the gut and intratumoural microbiota have been implicated in CRC tumourigenesis, with several pathobionts shown to contribute to tumour development and disease progression. In this study, we explored how bacterial co‐occurrence is related to the anti‐tumour immune response and clinical presentation of CRC. We found that the oral pathobionts *P. stomatis, F. nucleatum sensu lato* and *P. micra* were frequently co‐detected in tumours of CRC patients, and that their co‐occurrence was associated with an altered tumour immune microenvironment and a worse patient prognosis.

The co‐occurrence of *P. stomatis, F. nucleatum sensu lato* and *P. micra* in CRC tumours is consistent with previously described patterns of oral pathobiont enrichment in CRC. In our earlier study, we identified a faecal microbial network associated with CRC that included these taxa together with *Porphyromonas* spp., and another study reported this microbial signature, alongside *Akkermansia muciniphila*, as a four‐biomarker panel of CRC detection in tumour tissue [3, 19]. Furthermore, *P. stomatis* , *F. nucleatum* and *P. micra* have been associated with CRC tumours of the CMS1 subtype [20]. In line with this finding, we observed co‐occurrence of these bacteria predominantly in right‐sided CRC tumours with an immunogenic MSI phenotype (part of CMS1).

Having established the pattern of co‐occurrence, we further investigated its consequences on the anti‐tumour immune response. Tumours with high levels of *F. nucleatum sensu lato* in combination with *P. stomatis* and/or *P. micra* exhibited decreased numbers of FOXP3^+^ cells and CD68^+^ macrophages, alongside increased tumour epithelial expression of PD‐L1, compared to tumours with low bacterial levels or high levels of *F. nucleatum sensu lato* alone. These alterations in the tumour immune response were found mainly in the tumour centre, suggesting a localised immunosuppressive effect. The potential immunosuppressive effect of these bacteria was supported by the decreased survival observed in patients with bacterial co‐occurrence, given that the immune response is a well‐established positive prognostic factor in CRC [17]. FOXP3^+^ cells are linked to a poor prognosis in many cancer types, with CRC being one of the exceptions [32]. In this cohort, we previously reported a positive prognostic role for T regulatory cells in these patients [23]. Furthermore, we observed a strong positive correlation between FOXP3 and all other immune cell markers included in the analyses (both activating and inhibiting), which supports their concordant enrichment/depletion [27]. We therefore interpret our findings as indicative of an immunosuppressive effect.

Studies addressing the effect of bacterial co‐occurrence on tumour immunity remain limited. Previous work has demonstrated immunomodulatory effects of individual bacterial species. Intratumoural *F. nucleatum* abundance has been associated with lower densities of stromal CD3^+^ T cells [33], and contributes to an immunosuppressive microenvironment in CRC by diminishing antibacterial defence and anti‐tumour immunity [34]. Furthermore, *F. nucleatum* has been shown to stimulate PD‐L1 expression in CRC cells and to enhance the efficacy of PD‐L1 blockade [35, 36]. A parallel decrease in FOXP3^+^ T regulatory cells and increase in tumour cell PD‐L1 expression has previously been suggested in CRC [37], proposing a possible inverse relationship between these two. Additionally, a decreased infiltration by FOXP3^+^ cells has been reported in tumours with high levels of *F. nucleatum* in MSI‐H CRCs, accompanied by an increased proportion of M2‐polarised macrophages [38]. Consistently, Chen et al. demonstrated that *F. nucleatum* infection increased M2 macrophage polarisation in vitro and in vivo [39]. A shift in macrophage polarisation towards an M2 phenotype has also been suggested for *P. micra* [40]. In contrast, Borowsky et al. reported no association between *F. nucleatum* *sensu lato* and either M1 or M2 macrophages in CRC but instead observed reduced stromal lymphocyte infiltration [33]. Similarly, Kim et al. suggested that *F. nucleatum* may impair adaptive immunity in CRC, finding enrichment of exhausted cytotoxic T cells and T regulatory cells within the tumour microenvironment [41]. In this study, we find a general decrease in macrophages (CD68^+^) in the tumour centre of tumours with bacterial co‐occurrence, supporting a potential immunosuppressive effect. However, as CD68 is a pan‐macrophage marker, we were unable to discriminate between M1 and M2 macrophages. Although findings across studies are not entirely consistent, they collectively point towards a predominantly immunosuppressive effect by bacteria. Further studies are needed to elucidate the mechanisms underlying these potential interactions between bacterial species and the immune response.

*F. nucleatum* and *P. micra* have previously been shown to be highly correlated in the gut and in tumours of CRC patients [9, 42], to co‐aggregate, and to exhibit synergistic biofilm formation in vitro [43, 44]. The ability to form biofilms may account for the co‐occurrence observed in this study and may also suggest that biofilm organisation could be contributing to an immunosuppressed microenvironment. Furthermore, *F. animalis* has been shown to be enriched in invasive biofilms in CRC [45]. Although interesting, such associations remain speculative and should be confirmed in future studies.

Both *F. nucleatum* and *P. micra* have also been reported individually to be associated with adverse prognosis in CRC [8, 9]. A recent oncomicrobial profiling study further described three distinct oncomicrobial community subtypes including a cluster enriched in oral pathogens such as *F. nucleatum* and *P. micra* [46]. In line with our findings, this subtype was more frequent in right‐sided, high‐grade tumours, as well as in tumours harbouring *BRAF* mutations and an MSI phenotype and was associated with poorer prognosis in MSS CRCs. Previous studies, as well as the current study, have linked CRC‐associated bacteria to MSI CRCs, which are generally associated with higher immune infiltration and a favourable prognosis [47]. This may suggest that bacterial co‐occurrence identifies a subgroup of MSI CRCs with poorer prognosis. Further studies in larger patient cohorts are needed to strengthen this hypothesis.

One strength of the study is the comprehensive immune profiling, in which the distribution of multiple immune markers was evaluated across distinct tumour compartments. However, the use of TMAs may not fully capture the spatial heterogeneity of the immune cell infiltration throughout the entire tumour. Further studies using whole‐tissue sections could provide a more comprehensive assessment of the tumour immune microenvironment and further validate and extend these findings. The most pronounced alterations in the immune response associated with bacterial co‐occurrence were observed in the tumour centre. Additionally, microbial levels were assessed in fresh frozen tumour tissue, which has been shown to better preserve microbial content compared with FFPE samples [48].

Nevertheless, the findings of this study should be interpreted in the context of methodological limitations. Most previous research has focused on *F. nucleatum*, although much of the available findings predates its recent taxonomic reclassification. In particular, many studies referring to *F. nucleatum* do not distinguish between *F. nucleatum* *sensu stricto* and *sensu lato*. This may contribute to heterogeneity in reported findings across studies. The recent reclassification of *F. nucleatum* *sensu lato* into distinct species, including *F. animalis*, *F. nucleatum* , *F. vincentii* and *F. polymorphum* [22, 23], highlights the potential for species‐specific functions [21, 26]. The qPCR approach used in this study to target *F. nucleatum* lacks species‐level resolution. Recent evidence suggests that *F. animalis* is the most enriched *Fusobacterium* species in CRC, whereas other *Fusobacterium* species are detected at lower frequencies [45, 49]. In addition, our findings on bacterial co‐occurrence are based on associations and do not provide evidence of a direct spatial co‐localisation or the underlying mechanisms linking bacterial niches to an immunosuppressive tumour microenvironment.

Further experimental studies are needed to elucidate the biological behaviour of the bacterial cluster identified in this study. Particularly, it will be important to determine the spatial co‐localisation of the bacteria by performing FISH alone or combined with immunofluorescence. Co‐culture experiments could further explain the role of the bacterial cluster in CRC progression by assessing their effects on key tumour‐associated processes. Collectively, these investigations may provide further insight into whether the contribution of bacteria to CRC progression is better understood from a community or cluster perspective, rather than attributing specific effects solely to individual bacterial species. In the longer term, such work could support a shift from viewing the CRC‐associated microbiota as individual prognostic taxa towards defining functionally relevant microbial communities that inform risk stratification, patient selection for immunotherapy and microbiota‐targeted therapeutic strategies.

## 6 Conclusions

In conclusion, our findings demonstrate an association between *P. stomatis* , *F. nucleatum sensu lato* and *P. micra* in CRC tumours. Their co‐occurrence was associated with reduced numbers of FOXP3^+^ cells and macrophages and increased numbers of PD‐L1^+^ tumour cells in the tumour centre, suggesting a potential role by these bacteria in shaping the tumour immune microenvironment. Bacterial co‐occurrence was further associated with a poorer patient prognosis. Further mechanistic studies are needed to elucidate the interactions between microbes and the tumour immune microenvironment in CRC, which may have implications for therapeutic strategies.

## Author Contributions

**Marina Rubio Garcia:** conceptualization, investigation, writing – original draft, writing – review and editing, methodology, data curation, formal analysis, visualization. **Vicky Bronnec:** conceptualization, supervision, writing – review and editing, funding acquisition, methodology, resources, writing – original draft, visualization, investigation, formal analysis. **Maïwenn Kerdreux:** data curation, investigation, writing – review and editing. **William Rosenbaum:** investigation, formal analysis, writing – review and editing, methodology, data curation. **Thyra Löwenmark:** investigation, writing – review and editing, data curation. **Anna Löfgren‐Burström:** investigation, data curation, writing – review and editing, methodology. **Ingrid Ljuslinder:** writing – review and editing, resources, investigation. **Sofia Edin:** conceptualization, methodology, supervision, funding acquisition, visualization, writing – review and editing, writing – original draft, resources, formal analysis, data curation. **Richard Palmqvist:** conceptualization, supervision, resources, project administration, writing – review and editing, visualization, methodology, funding acquisition, investigation.

## Funding

This work was supported by the Swedish Cancer Society, 23 2901 Pj. Lion's Cancer Research Foundation, LP 24‐2355. Cancer Research Foundation in Northern Sweden, AMP 25‐1194, AMP‐24 1155. Cutting edge grant from the County Council of Västerbotten, RV‐1006492. Regional agreement between Umeå University and Region Västerbotten (ALF), RV‐1034892.

## Ethics Statement

The study protocol was approved by the Ethics Committee of Umeå University/Regional Ethics Review Board (2014/321‐31, 2016/219‐31 and amendment 2019‐00566).

## Consent

Informed consent was obtained from all individual participants included in the study.

## Conflicts of Interest

The authors declare no conflicts of interest.

## Supporting information


**Figure S1:** qPCR for detection of *Fusobacterium* species. Shown are representative qPCR amplification curves illustrating detection of *F. animalis (DSM 19367)*, *F. nucleatum (DSM 15643)*, *F. vincentii (DSM 507)* and *F. polymorphum (DSM 20482)*. Each curve corresponds to the amplification profile of one species‐specific target. The *x*‐axis shows the PCR cycle number, and the y‐axis shows the fluorescence signal (ΔRn), reflecting the accumulation of amplified product.
**Table S1:** Univariable and multivariable COX proportional hazard analyses of 5‐year cancer‐specific survival according to bacterial co‐occurrence.
**Figure S2:** Multispectral imaging of immune markers in the tumour centre of CRC specimens according to different bacterial co‐occurrence groups. Shown are representative multispectral images of tumour microarray cores (1 mm) stained with (a) *Panel I*; CD8 (red), T‐bet (white), FoxP3 (orange), CD20 (yellow), CD68 (cyan) and cytokeratin (magenta), and (b) *Panel II*; CTLA‐4 (red), PD‐L1 (yellow), PD‐1 (cyan), FoxP3 (orange) and cytokeratin (magenta). The left column displays tumours classified within the low bacterial co‐occurrence group, while the right column displays tumours classified within the polymicrobial high co‐occurrence group. Images were acquired at x20 magnification. Nuclei were counterstained with DAPI.

## Data Availability

The data that support the findings of this study are available on request from the corresponding author. The data are not publicly available due to privacy or ethical restrictions.

## References

[1] International Agency for Research on Cancer , “Cancer Today. Global Cancer Observatory,” (2024), https://gco.iarc.who.int/media/globocan/factsheets/cancers/41‐colorectum‐fact‐sheet.pdf.

[2] S. M. Ahmad Kendong , R. A. Raja Ali , K. N. M. Nawawi , H. F. Ahmad , and N. M. Mokhtar , “Gut Dysbiosis and Intestinal Barrier Dysfunction: Potential Explanation for Early‐Onset Colorectal Cancer,” Frontiers in Cellular and Infection Microbiology 11 (2021): 744606, 10.3389/fcimb.2021.744606. PMC8710575 34966694

[3] M. A. Osman , H. m. Neoh , N. S. Ab Mutalib , et al., “ *Parvimonas micra* , *Peptostreptococcus stomatis* , Fusobacterium Nucleatum and *Akkermansia muciniphila* as a Four‐Bacteria Biomarker Panel of Colorectal Cancer,” Scientific Reports 11 (2021): 2925, 10.1038/s41598-021-82465-0. PMC7859180 33536501

[4] J. Wirbel , P. T. Pyl , E. Kartal , et al., “Meta‐Analysis of Fecal Metagenomes Reveals Global Microbial Signatures That Are Specific for Colorectal Cancer,” Nature Medicine 25 (2019): 679–689, 10.1038/s41591-019-0406-6. PMC7984229 30936547

[5] Z. Dai , O. O. Coker , G. Nakatsu , et al., “Multi‐Cohort Analysis of Colorectal Cancer Metagenome Identified Altered Bacteria Across Populations and Universal Bacterial Markers,” Microbiome 6 (2018): 70, 10.1186/S40168-018-0451-2. PMC5896039 29642940

[6] C. Gur , Y. Ibrahim , B. Isaacson , et al., “Binding of the Fap2 Protein of *fusobacterium nucleatum* to Human Inhibitory Receptor TIGIT Protects Tumors From Immune Cell Attack,” Immunity 42 (2015): 344–355, 10.1016/j.immuni.2015.01.010. PMC4361732 25680274

[7] L. Flanagan , J. Schmid , M. Ebert , et al., “ *Fusobacterium nucleatum* Associates With Stages of Colorectal Neoplasia Development, Colorectal Cancer and Disease Outcome,” European Journal of Clinical Microbiology & Infectious Diseases 33 (2014): 1381–1390, 10.1007/s10096-014-2081-3. 24599709

[8] L. Zhao , X. Zhang , Y. Zhou , et al., “ *Parvimonas micra* Promotes Colorectal Tumorigenesis and Is Associated With Prognosis of Colorectal Cancer Patients,” Oncogene 41, no. 36 (2022): 4200–4210, 10.1038/s41388-022-02395-7. PMC9439953 35882981

[9] T. Löwenmark , A. Löfgren‐Burström , C. Zingmark , et al., “Tumour Colonisation of *Parvimonas micra* Is Associated With Decreased Survival in Colorectal Cancer Patients,” Cancers (Basel) 14 (2022): 14, 10.3390/cancers14235937. PMC9736682 36497419

[10] M. Kerdreux , S. Edin , T. Löwenmark , et al., “ *Porphyromonas gingivalis* in Colorectal Cancer and Its Association to Patient Prognosis,” Journal of Cancer 14 (2023): 1479–1485, 10.7150/jca.83395. PMC10266249 37325051

[11] X. Wang , Y. Jia , L. Wen , et al., “ *Porphyromonas gingivalis* Promotes Colorectal Carcinoma by Activating the Hematopoietic NLRP3 Inflammasome,” Cancer Research 81 (2021): 2745–2759, 10.1158/0008-5472.CAN-20-3827. 34003774

[12] P. Huang , F. Ji , A. H. K. Cheung , et al., “ *Peptostreptococcus stomatis* Promotes Colonic Tumorigenesis and Receptor Tyrosine Kinase Inhibitor Resistance by Activating ERBB2‐MAPK,” Cell Host & Microbe 32 (2024): 1365–1379.e10, 10.1016/j.chom.2024.07.001. 39059397

[13] S. S. Jiang , Y. le Xie , X. Y. Xiao , et al., “ *Fusobacterium nucleatum* ‐Derived Succinic Acid Induces Tumor Resistance to Immunotherapy in Colorectal Cancer,” Cell Host & Microbe 31 (2023): 781–797.e9, 10.1016/j.chom.2023.04.010. 37130518

[14] Y. Liu , C. C. Wong , Y. Ding , et al., “ *Peptostreptococcus anaerobius* Mediates Anti‐PD1 Therapy Resistance and Exacerbates Colorectal Cancer via Myeloid‐Derived Suppressor Cells in Mice,” Nature Microbiology 9 (2024): 1467–1482, 10.1038/S41564-024-01695-W. PMC11153135 38750176

[15] X. Wang , Y. Fang , W. Liang , et al., “ *Fusobacterium nucleatum* Facilitates Anti‐PD‐1 Therapy in Microsatellite Stable Colorectal Cancer,” Cancer Cell 42 (2024): 1729–1746.e8, 10.1016/j.ccell.2024.08.019. 39303724

[16] J. L. Galeano Niño , H. Wu , K. D. LaCourse , et al., “Effect of the Intratumoral Microbiota on Spatial and Cellular Heterogeneity in Cancer,” Nature 611, no. 7937 (2022): 810–817, 10.1038/s41586-022-05435-0. PMC9684076 36385528

[17] J. Galon , A. Costes , F. Sanchez‐Cabo , et al., “Type, Density, and Location of Immune Cells Within Human Colorectal Tumors Predict Clinical Outcome,” Science 313 (2006): 1960–1964, 10.1126/science.1129139. 17008531

[18] K. E. Pauken , J. A. Torchia , A. Chaudhri , A. H. Sharpe , and G. J. Freeman , “Emerging Concepts in PD‐1 Checkpoint Biology,” Seminars in Immunology 52 (2021): 101480, 10.1016/j.smim.2021.101480. PMC8545711 34006473

[19] T. Löwenmark , L. Köhn , T. Kellgren , et al., “ *Parvimonas micra* Forms a Distinct Bacterial Network With Oral Pathobionts in Colorectal Cancer Patients,” Journal of Translational Medicine 22 (2024): 947, 10.1186/s12967-024-05720-8. PMC11487773 39420333

[20] R. V. Purcell , M. Visnovska , P. J. Biggs , S. Schmeier , and F. A. Frizelle , “Distinct Gut Microbiome Patterns Associate With Consensus Molecular Subtypes of Colorectal Cancer,” Scientific Reports 7 (2017): 7, 10.1038/s41598-017-11237-6. PMC5599497 28912574

[21] M. A. Zepeda‐Rivera , F. E. Dewhirst , S. Bullman , and C. D. Johnston , “Addressing Controversy in Fusobacterium Nomenclature: What Exactly Does “ *F. nucleatum* ” Refer to?,” Gut Microbes 17 (2025): 17, 10.1080/19490976.2025.2514797. PMC12143674 40464118

[22] T. Löwenmark , A. Löfgren‐Burström , C. Zingmark , et al., “ *Parvimonas micra* as a Putative Non‐Invasive Faecal Biomarker for Colorectal Cancer,” Scientific Reports 10 (2020): 15250, 10.1038/s41598-020-72132-1. PMC7499209 32943695

[23] S. Edin , B. Gylling , X. Li , et al., “Opposing Roles by KRAS and BRAF Mutation on Immune Cell Infiltration in Colorectal Cancer—Possible Implications for Immunotherapy,” British Journal of Cancer 130, no. 1 (2023): 143–150, 10.1038/s41416-023-02483-9. PMC10781968 38040818

[24] M. Dreier , H. Berthoud , N. Shani , D. Wechsler , and P. Junier , “SpeciesPrimer: A Bioinformatics Pipeline Dedicated to the Design of qPCR Primers for the Quantification of Bacterial Species,” PeerJ 8 (2020): e8544, 10.7717/PEERJ.8544. PMC7034379 32110486

[25] V. Bronnec and O. A. Alexeyev , “In Vivo Model of Propionibacterium (Cutibacterium) spp. Biofilm in *Drosophila melanogaster* ,” Anaerobe 72 (2021): 102450, 10.1016/J.ANAEROBE.2021.102450. 34619359

[26] W. Rosenbaum , M. R. Garcia , A. Löfgren‐Burström , et al., “Full‐Length 16S rRNA Nanopore Sequencing Enables Species Resolution of Fusobacterium Associated With Colorectal Cancer,” Gut Microbes 18 (2026): 2656004, 10.1080/19490976.2026.2656004. PMC13078227 41963777

[27] S. Edin , B. Gylling , X. Li , et al., “Differential Distribution of Immune Checkpoints Across Molecular Subtypes of Colorectal Cancer,” Oncoimmunology 14 (2025): 14, 10.1080/2162402X.2025.2546406. PMC12372490 40838947

[28] T. Takahashi , T. Tagami , S. Yamazaki , et al., “Immunologic Self‐Tolerance Maintained by Cd25+Cd4+Regulatory T Cells Constitutively Expressing Cytotoxic T Lymphocyte–Associated Antigen 4,” Journal of Experimental Medicine 192 (2000): 303–310, 10.1084/JEM.192.2.303. PMC2193248 10899917

[29] G. R. Kim and J. M. Choi , “Current Understanding of Cytotoxic T Lymphocyte Antigen‐4 (CTLA‐4) Signaling in T‐Cell Biology and Disease Therapy,” Molecules and Cells 45 (2022): 513–521, 10.14348/MOLCELLS.2022.2056. PMC9385567 35950451

[30] D. Masopust , A. Awasthi , R. Bosselut , et al., “Guidelines for T Cell Nomenclature,” Nature Reviews Immunology 26, no. 4 (2025): 298–313, 10.1038/s41577-025-01238-2. 41254224

[31] M. Charpentier , S. Spada , S. J. Van Nest , et al., “Radiation Therapy‐Induced Remodeling of the Tumor Immune Microenvironment,” Seminars in Cancer Biology 86 (2022): 737–747, 10.1016/j.semcancer.2022.04.003. 35405340

[32] B. Shang , Y. Liu , S. J. Jiang , et al., “Prognostic Value of Tumor‐Infiltrating FoxP3+ Regulatory T Cells in Cancers: A Systematic Review and Meta‐Analysis,” Scientific Reports 5, no. 1 (2015): 15179, 10.1038/srep15179. PMC4604472 26462617

[33] J. Borowsky , K. Haruki , M. C. Lau , et al., “Association of *Fusobacterium nucleatum* With Specific T Cell Subsets in the Colorectal Carcinoma Microenvironment,” Clinical Cancer Research 27 (2021): 2816–2826, 10.1158/1078-0432.CCR-20-4009. PMC8127352 33632927

[34] R. Heregger , F. Huemer , R. Greil , et al., “Impact of *Fusobacterium nucleatum* on Immune Cell Interactions and Gene Expression in Colorectal Cancer: A Retrospective Cohort Study,” Frontiers in Immunology 16 (2025): 1629014, 10.3389/FIMMU.2025.1629014/FULL. PMC12460317 41019049

[35] Y. Gao , D. Bi , R. Xie , et al., “ *Fusobacterium nucleatum* Enhances the Efficacy of PD‐L1 Blockade in Colorectal Cancer,” Signal Transduction and Targeted Therapy 6, no. 1 (2021): 398, 10.1038/s41392-021-00795-x. PMC8602417 34795206

[36] C. Duizer , M. Salomons , M. van Gogh , et al., “ *Fusobacterium nucleatum* Upregulates the Immune Inhibitory Receptor PD‐L1 in Colorectal Cancer Cells via the Activation of ALPK1,” Gut Microbes 17 (2025): 17, 10.1080/19490976.2025.2458203. PMC11784648 39881579

[37] Y. Masugi , R. Nishihara , J. Yang , et al., “Tumour CD274 (PD‐L1) Expression and T Cells in Colorectal Cancer,” Gut 66 (2017): 1463–1473, 10.1136/gutjnl-2016-311421. PMC5097696 27196573

[38] J. A. Lee , S. Y. Yoo , H. J. Oh , et al., “Differential Immune Microenvironmental Features of Microsatellite‐Unstable Colorectal Cancers According to *Fusobacterium nucleatum* Status,” Cancer Immunology, Immunotherapy 70 (2021): 47–59, 10.1007/s00262-020-02657-x. PMC10991456 32623478

[39] T. Chen , Q. Li , J. Wu , et al., “ *Fusobacterium nucleatum* Promotes M2 Polarization of Macrophages in the Microenvironment of Colorectal Tumours via a TLR4‐Dependent Mechanism,” Cancer Immunology, Immunotherapy 67, no. 10 (2018): 1635–1646, 10.1007/S00262-018-2233-X. PMC11028377 30121899

[40] D. K. Nguyen , M. J. Kang , S. J. Oh , et al., “ *Parvimonas micra* ‐Polarized M2‐Like Tumor‐Associated Macrophages Accelerate Colorectal Cancer Development via IL‐8 Secretion,” Animal Cells and Systems 29 (2024): 24–34, 10.1080/19768354.2024.2442401. PMC11703389 39777026

[41] H. S. Kim , C. G. Kim , W. K. Kim , et al., “ *Fusobacterium nucleatum* Induces a Tumor Microenvironment With Diminished Adaptive Immunity Against Colorectal Cancers,” Frontiers in Cellular and Infection Microbiology 13 (2023): 1101291, 10.3389/FCIMB.2023.1101291/TEXT. PMC10028079 36960042

[42] J. Yu , Q. Feng , S. H. Wong , et al., “Metagenomic Analysis of Faecal Microbiome as a Tool Towards Targeted Non‐Invasive Biomarkers for Colorectal Cancer,” Gut 66 (2017): 70–78, 10.1136/gutjnl-2015-309800. 26408641

[43] A. Horiuchi , E. Kokubu , T. Warita , and K. Ishihara , “Synergistic Biofilm Formation by Parvimonas Micra and *Fusobacterium nucleatum* ,” Anaerobe 62 (2020): 102100, 10.1016/j.anaerobe.2019.102100. 31521732

[44] A. V. Robinson and E. Allen‐Vercoe , “Strain Specificity in Fusobacterial Co‐Aggregation With Colorectal Cancer‐Relevant Species,” Anaerobe 82 (2023): 102758, 10.1016/j.anaerobe.2023.102758. 37423597

[45] J. Queen , Z. Cing , H. Minsky , et al., “ *Fusobacterium nucleatum* Is Enriched in Invasive Biofilms in Colorectal Cancer,” npj Biofilms Microbiomes 11 (2025): 81, 10.1038/s41522-025-00717-7. PMC12092649 40394001

[46] D. Mouradov , P. Greenfield , S. Li , et al., “Oncomicrobial Community Profiling Identifies Clinicomolecular and Prognostic Subtypes of Colorectal Cancer,” Gastroenterology 165 (2023): 104–120, 10.1053/J.GASTRO.2023.03.205. 36933623

[47] S. Popat , R. Hubner , and R. S. Houlston , “Systematic Review of Microsatellite Instability and Colorectal Cancer Prognosis,” Journal of Clinical Oncology 23 (2005): 609–618, 10.1200/JCO.2005.01.086. 15659508

[48] A. Borgognone , G. Serna , M. Noguera‐Julian , et al., “Performance of 16s Metagenomic Profiling in Formalin‐Fixed Paraffin‐Embedded Versus Fresh‐Frozen Colorectal Cancer Tissues,” Cancers 13 (2021): 5421, 10.3390/CANCERS13215421/S1. PMC8582506 34771584

[49] M. Zepeda‐Rivera , S. S. Minot , H. Bouzek , et al., “A Distinct *Fusobacterium nucleatum* Clade Dominates the Colorectal Cancer Niche,” Nature 628 (2024): 424–432, 10.1038/s41586-024-07182-w. PMC11006615 38509359

