# Supplementary material for: Intratumoural Bacterial Co‐Occurrence is Associated With an Altered Tumour Immune Microenvironment and Poor Prognosis in Colorectal Cancer
Source: Cancer Med. 2026 Oct 29;15(11):e72391. doi: 10.1002/cam4.72391 (PMC13717388; doi:10.1002/cam4.72391)
Supplement: Supplementary file 1 [file CAM4-15-e72391-s001.docx]

**Supplementary Figure S1**

**Figure S1:** qPCR for detection of *Fusobacterium* species. Shown are representative qPCR amplification curves illustrating detection of *F. animalis (DSM 19367)*, *F. nucleatum (DSM 15643)*, *F. vincentii (DSM 507)*, and *F. polymorphum (DSM 20482)*. Each curve corresponds to the amplification profile of one species-specific target. The x-axis shows the PCR cycle number, and the y-axis shows the fluorescence signal (ΔRn), reflecting the accumulation of amplified product.

**Supplementary Figure S2**

**Supplementary Figure S2: Multispectral imaging of immune markers in the tumour centre of CRC specimens according to different bacterial co-occurrence groups.** Shown are representative multispectral images of tumour microarray cores (1 mm) stained with (a) *Panel I*; CD8 (red), T-bet (white), FoxP3 (orange), CD20 (yellow), CD68 (cyan), and cytokeratin (magenta), and (b) *Panel II*; CTLA-4 (red), PD-L1 (yellow), PD-1 (cyan), FoxP3 (orange), and cytokeratin (magenta). The left column displays tumours classified within the low bacterial co-occurrence group, while the right column displays tumours classified within the polymicrobial high co-occurrence group. Images were acquired at x20 magnification. Nuclei were counterstained with DAPI.

**Supplementary Table S1**

|  | **COX univariable analysis** | | |  |
| --- | --- | --- | --- | --- |
|  | **Hazard ratio** | **95.0% CI** | **p-value** |  |
| **Sex**  Male  Female | 1  0.82 | 0.41 – 1.65 | 0.584 |  |
| **Age**  ≤ 59  60-69  70-79  ≥ 80 | 1  0.42  0.51  0.56 | 0.18 – 1.02  0.21 – 1.22  0.18 – 1.80 | 0.055  0.129  0.333 |  |
| **Localisation**  Right colon  Left colon  Rectum | 1  0.80  0.98 | 0.24 – 2.75  0.42 – 2.27 | 0.731  0.954 |  |
| **Stage**  I-II  III  IV | 1  6.26  34.07 | 2.04 – 19.20  11.26 – 103.0 | 0.001*  <0.001* |  |
| **Tumour type**  Not mucinous  Mucinous | 1  2.10 | 0.86 – 5.15 | 0.103 |  |
| **Tumour grade**  Low  High | 1  4.80 | 2.24 – 10.27 | <0.001* |  |
| ***KRAS***  Wild type  Mutant | 1  1.21 | 0.55 – 2.66 | 0.638 |  |
| ***BRAF***  Wild type  Mutant | 1  1.27 | 0.44 – 3.69 | 0.660 |  |
| **MMR status**  MSS  MSI | 1  0.75 | 0.18 – 3.18 | 0.698 |  |
| **Pre-operative radiotherapy**  No  Yes | 1  1.73 | 0.87 – 3.45 | 0.122 |  |
| **Bacterial co-occurrence**  Low  *Fuso4* high only  Polymicrobial high | 1  1.55  4.09 | 0.31 – 7.68  1.37 – 12.19 | 0.592  0.012* |  |
| *Indicates statistically significant p-values <0.05. Abbreviations: *Fuso4*, *F. nucleatum* *sensu lato.* | | | | |

**Table S1: Univariable and multivariable COX proportional hazard analyses of 5-year cancer-specific survival according to bacterial co-occurrence.**
